# Adolescents’ physical activity and sedentary behaviour in Indonesia during the COVID-19 pandemic: a qualitative study of mothers’ perspectives

**DOI:** 10.1186/s12889-021-11931-1

**Published:** 2021-10-15

**Authors:** Fitria Dwi Andriyani, Stuart J. H. Biddle, Katrien De Cocker

**Affiliations:** 1 Physically Active Lifestyles Research Group (USQ PALs), Centre for Health Research, University of Southern Queensland, Springfield Central, 4300 Australia; 2 Department of Sports Education, Faculty of Sports Science, Yogyakarta State University, Yogyakarta, 55281 Indonesia; 3 Department of Movement and Sports Sciences, Faculty of Medicine and Health Sciences, Ghent University, Ghent, B9000 Belgium

**Keywords:** COVID-19, Health, Youth, Physical activity, Screen time, Sedentary behaviour

## Abstract

**Background:**

Socio-behavioural adaptations during the COVID-19 pandemic may have significantly affected adolescents’ lifestyle. This study aimed to explore possible reasons affecting changes in physical activity and sedentary behaviour in Indonesian adolescents during the pandemic based on mothers’ perspectives.

**Methods:**

We recruited parents (*n* = 20) from the Yogyakarta region of Indonesia (July–August 2020) using purposive and snowball sampling. Individual interviews were audio-recorded, transcribed verbatim and anonymised. Data were imported into NVivo software for a reflexive thematic analysis.

**Results:**

The interviews lasted between 38 and 113 min (*M* = 65 min). Participants’ age ranged between 36 and 54 years (M = 42.6 years). Participants’ children ranged in age from 12 to 15 years (M = 13.7 years, female: 9, male: 11). Themes related to changes in physical activity during the pandemic were 1) self-determination and enjoyment, 2) supports from others, and 3) physical activity facilities and equipment. Themes related to changes in sedentary behaviour during the pandemic included 1) educational demands, 2) psychological effects due to the pandemic, 3) devices and internet availability, 4) parental control, and 5) social facilitators.

**Conclusions:**

During the pandemic, mothers perceived their children to be less active and using more screen-based devices, either for educational or recreational purposes, compared to before. The present themes might be useful when developing interventions and policies promoting physical activity and reducing sedentary behaviour in adolescents. Interventions could, for example, consider increasing parents’ and adolescents’ awareness on current activity guidelines, providing education on healthier recreational screen time, and involving parents, peers, and teachers. Increasing the accessibility of physical activity facilities and equipment, making use of adolescents’ favourite program and social media for interventions, and providing activities that are fun and enjoyable may also important.

**Supplementary Information:**

The online version contains supplementary material available at 10.1186/s12889-021-11931-1.

## Background

It has been widely known that maintaining a sufficient level of physical activity and limiting sedentary behaviour are crucial to support both physical and mental health in youth [1–4]. For young people, physical activity was associated with better adiposity profiles, cardiometabolic biomarkers, physical fitness, bone health, cognitive function and academic achievement [1, 3]. In contrast, sedentary behaviour can be associated with detrimental health outcomes, including lower fitness and sleep quality, unfavourable BMI, higher cardiometabolic risk, and lower psychosocial health [3, 5, 6]. The 2020 World Health Organization (WHO) guidelines recommended that children and adolescents (5–17 years) accumulate an average of 60 min of moderate-to-vigorous physical activity (MVPA) every day and limit the duration of recreational screen time to achieve positive health outcomes [7].

In December 2019, the first case of severe acute respiratory syndrome coronavirus 2 (SARS-CoV-2) (COVID-19) was identified in Wuhan City, China [8]. Shortly after, the disease began to spread rapidly across the world. Social distancing and quarantining/isolating have become common practices as measures to hamper the spread of the outbreak. The general public was encouraged to stay at home and to do only essential outdoor activities, such as grocery shopping. Moreover, as of March 26, 2020, school closures have been enforced nationwide in 166 countries, affecting over 80% of enrolled students (more than 1.4 billion young people) worldwide [9].

In Indonesia, the first COVID-19 case was confirmed on the 2nd of March 2020 [10]. In Yogyakarta region, emergency response status was implemented from March to August 2020 [11]. Even though there was no restriction to do exercise outside the house, communities in the region were encouraged to limit their mobility and public sports facilities were closed during those time [12–14]. School closures have also been enforced from the end of March 2020 [15]. Since then, students have been studying online at home.

These changed circumstances may have affected young people’s lifestyle profoundly, including their physical activity and sedentary behaviour. Even before the COVID-19 pandemic, with the freedom to do activities and access to public amenities, more than three-quarters (81%) of adolescents did not meet the WHO physical activity guidelines [16]. In a large review of population studies, Thomas et al. (2020) reported that over 50% of young people exceeded 2 h/day of recreational screen time, with screen time averaging 3.6 h/day [17]. The COVID-19 outbreak may have exacerbated these trends. Preliminary studies suggested that socio-behavioural adaptations during the COVID-19 pandemic, including social distancing and isolating, have caused a significant reduction in physical activity [18–20], an increased engagement in sedentary behaviour [19, 21, 22], and disrupted sleep schedule [21, 23] among children and adolescents. In the long term, this may accumulate to cause serious health problems.

Previous COVID-19 studies on physical activity and sedentary behaviour have investigated the effects of the pandemic on the levels of participation in both behaviours [18–20]. Other studies have focused on the effect of physical activity and sedentary behaviour on health outcomes during the pandemic [24, 25]. Investigations on these topics have largely employed cross-sectional designs using quantitative questionnaire data [20, 26, 27]; these have known limitations, including recall and social-desirability bias [28]. Meanwhile, research on the reasons for changes in adolescents’ physical activity and sedentary behaviour during the pandemic by using qualitative approaches are not yet available. There is also a paucity of data from low-to-middle income countries (LMICs), which is often suffering from a high population prevalence of physical inactivity and sedentary behaviour. A large scoping review revealed that in Indonesia, only 12.2–52.3% of youth report sufficient physical activity and between 24.5–33.8% have sedentary behaviour levels ≥3 h/day [29].

This study, therefore, aimed to investigate reasons affecting changes in physical activity and sedentary behaviour in Indonesian adolescents during the COVID-19 pandemic based on mothers’ perspectives by using a qualitative approach. Parental views were explored as they may provide important information on the determinants of children’s behaviour [30] and at the same time provide a narrative on the contexts of children’s behaviour. Investigating adolescents’ physical activity and sedentary behaviour during the pandemic through their parents’ perception has become a common practice in existing studies [20, 22, 26, 27]. A previous study indicated that parents may influence children’s physical activity and sedentary behaviour during the pandemic [22], hence we sought to examine the underlying factors further based on the perspectives of mothers.

## Methods

This study conformed to the Standards for Reporting Qualitative Research (SRQR) [31] (See Table S1). Grounded in the pragmatism paradigm, we chose a qualitative approach to achieve our research objectives. Pragmatism is a paradigm that breaks through the dichotomy between post-positivism and constructivism [32]. Pragmatists position the research objectives as central and implement all sound methods to answer the problems [33]. Methods for collecting and analysing data were selected for their ability to provide an understanding of the research problem without a philosophical commitment to specific paradigms [34].

This study aimed to explore parents’ perspective of underlying reasons thought to affect changes in physical activity and sedentary behaviour in adolescents during the COVID-19 pandemic. We believe that a qualitative approach is the most appropriate method for achieving those research objectives as it can facilitate in-depth insights, and more multi-layered understanding and interpretation in investigating real-world problems [35, 36].

### Participants

Participants were recruited by using purposive and snowball sampling [37], to ensure that the potential participants fulfill the recruitment criteria and to make the recruitment process more efficient, especially during the COVID-19 pandemic. To participate in the study, participants had to be parents or caregivers that supervised at least one adolescent child who studied at junior high school level and lived in the Yogyakarta region of Indonesia.

All prospective participants received research information packs that explained the details of the study. The packs were sent to participants either through paper-based mail, e-mail or as a WhatsApp attachment. Parents that agreed to participate were asked to complete an online questionnaire that consisted of a consent form and sociodemographic questions, including age, the highest education level, employment status, and child’s age. The online questionnaire was hosted on the secure host University online survey platform.

Twenty mothers agreed to participate in the study. Taking into account the heterogeneous nature of the participants’ sociodemographic data, the balance between male and female children of the participants, and the extensive research questions, we believe that this number is sufficient (see [38]).

While “data saturation” has become a standard criterion in determining the sample size in thematic analysis research, we did not adopt this practice because of its incompatibility with the reflexive thematic analysis approach that we use in this study (see [38, 39]). In the reflexive thematic analysis approach, coding is an “open, fluid, organic, and recursive practice”, where codes are never fixed and “can evolve, be renamed, split into several codes, collapsed together with other codes, and even be abandoned” [38]. Moreover, coding can also move from the surface (semantic) meaning into more interpretive (latent) meaning as researchers get more engage with data [38].

### Interview

Semi-structured one-on-one interviews were conducted by the lead author using an interview guide. As the interview was semi-structured, the interviewer could ask questions beyond those in the interview guide to probe for more information and context [40].

Interviews were conducted primarily by mobile phone, either using conventional phone call, WhatsApp call or Zoom call as preferred by participants (*n* = 17). Three participants asked to do face-to-face interviews in the interviewer’s house due to problems with telephone connections and convenience reasons. Face-to-face interviews were conducted by strictly following the COVID-19 health and safety protocol, including wearing masks, and social distancing.

Interviews were conducted in July and August 2020 and were audio-recorded by using Olympus Digital Voice Recorder (DS-3500) and Olympus Boundary Microphone (ME33). Each interview was started with warm-up questions to build rapport with the participant. The interviewer briefly explained the study and how the privacy and confidentiality of participants were protected. Afterwards, the interviewer explained the outline of the interview and offered opportunities for the participants to ask any questions. Participants could ask to stop the interview at any time and they could refuse to answer any questions. Participants were offered an opportunity to review and revise their statement on the transcript and were given 3 days from receiving the transcript to change their statement. The interviews lasted between 38 and 113 min (*M* duration = 65 min). Upon completion of the interview, participants were offered a grocery voucher valued at AUD ($) 10 as a token of appreciation for their time.

### Data analysis

All interview recordings were transcribed verbatim and anonymised. Transcripts were imported into NVivo software (Version 12 Pro, QSR International, Victoria, Australia).

This study used a reflexive thematic analysis approach to analyse data. This is a method for identifying, analysing and reporting patterns or themes within data, where analysis is done after deeply engaging with the data; and that themes are actively generated by the researcher as themes do not passively emerge from data [39, 41]. Themes can be described as specific patterns based on interpretation across the dataset with a central organising concept [39, 42]. In the reflexive thematic analysis, researcher subjectivity is understood as a resource rather than “a potential threat to knowledge production”, which reflect the nature of qualitative research that is creative, reflexive and subjective [39].

The transcripts were analysed by following phases in the reflexive thematic analysis approach as follows: 1) Familiarisation with the data, 2) Coding, 3) Generating initial themes, 4) Reviewing themes, 5) Defining and naming themes, and 6) Writing up [39, 43]. Firstly, the lead author (FDA), who interviewed the participants, became familiarised with and immersed in, the dataset by listening to the interview recordings and reading the transcripts. After that, she conducted the data coding using the NVivo software. Codes were generated on both semantic and latent level to enable the researcher to explore both the surface and the underlying meaning from participants’ voices [41, 43].

After data coding, the first author then generated initial themes by using the inductive approach. The strength of this is to prevent the theme’s generation to be driven by the pre-existing theoretical interest of the researcher [41] that may interfere with the analysis and interpretation of data. The researcher produced themes without trying to connect them to previously established theories as this may create some barriers for the researcher to see something “new” and “different”. Afterwards, all authors critically reviewed themes, including defining and naming themes. Data were analysed between November 2020 and March 2021.

### Researcher characteristics and reflexivity

The lead author is a PhD candidate and a lecturer at a university Faculty of Sports Science, with previous experience of qualitative research methods. She adopted the pragmatism paradigm which influences her way of thinking in looking for the most appropriate methods to achieve her research objectives [33]. Her philosophical paradigm shapes her position that she does not want to be tied to, or has a philosophical commitment to specific paradigms [34].

The lead author conducted all interviews and data analysis. As some of the participants were recruited from personal contacts, this may influence the interview process and how those participants responded to the interview questions. The pre-established connection between the lead author and those participants may facilitate the participants to tell their true stories but may also provide some barriers to reveal stories due to social desirability bias. The study information sheet may influence participants’ responses to research questions. Nevertheless, the lead author always explained to the participants the need to tell their real situation and perspectives and tried to build good rapport and conversation throughout the interviews. Sharing and living in a similar cultural background in the Yogyakarta region of Indonesia may also provide some degree of connectedness between the lead author and the participants that make participants feel comfortable to share their stories and may facilitate the smoothness of the interviews.

To maintain reflexivity in interpreting data, the lead author discussed her thoughts with the second and third authors to check for any personal biases. These authors are experienced senior academics and research supervisors in exercise psychology and public health.

## Results

**Fig. 1:**
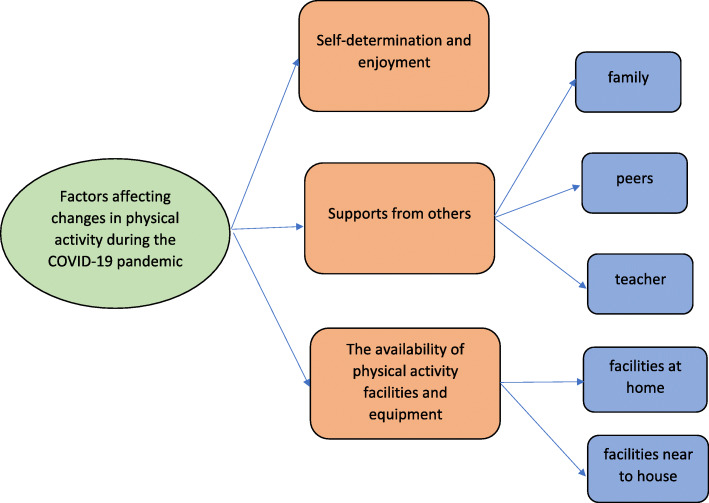
Factors affecting adolescents’ physical activity during the COVID-19 pandemic

**Fig. 2:**
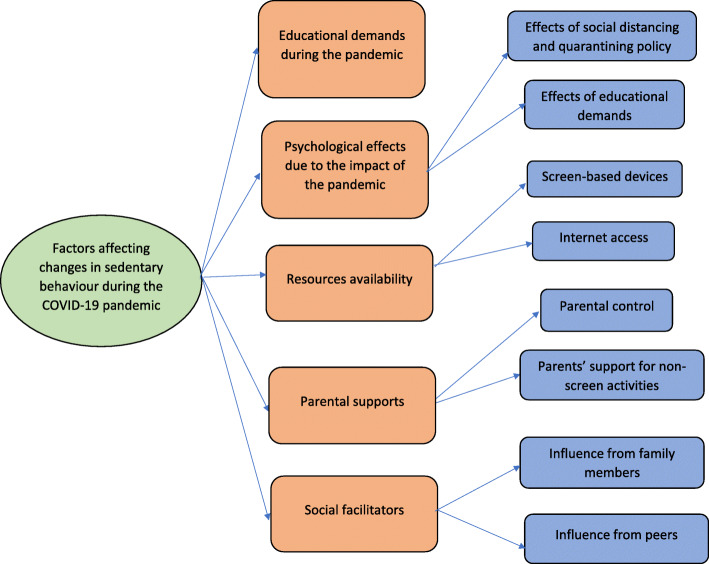
Factors affecting adolescents’ sedentary behaviour during the COVID-19 pandemic

The characteristics of the sample are presented in Table 1. Based on data analysis, we generated three themes related to changes in physical activity during the COVID-19 pandemic: 1) self-determination and enjoyment to do physical activity, 2) supports from others, and 3) the availability of physical activity facilities and equipment. The thematic map of these themes can be seen in Fig. 1. Meanwhile, five themes were identified that related to changes in sedentary behaviour: 1) educational demands during the pandemic, 2) psychological effects due to the impact of the pandemic, 3) devices and internet availability, 4) parental control, and 5) social facilitators. The thematic map of these themes can be seen in Fig. 2.

**Table 1:** Characteristics of the interview sample (*N* = 20)

Variables	Total	%
Age (Years)	*M* = 42.6	*SD* = 6.17
35–40	12	60%
41–45	2	10%
46–50	2	10%
51–55	4	20%
Education		
Year 6	1	5%
Year 9	2	10%
Year 12	8	40%
Bachelor degree	7	35%
Doctoral degree	2	10%
Occupation before the pandemic		
Labourer	3	15%
Lecturer	2	10%
Housemaid	2	10%
Tailor	2	10%
Teacher	6	30%
Entrepreneur	2	10%
On-Call Driver	1	5%
Housewife	1	5%
Multi-Occupation	1	5%
Occupation during the pandemic		
Labourer	3	15%
Lecturer	2	10%
Housemaid	1	5%
Tailor	2	10%
Teacher	6	30%
Entrepreneur	3	15%
Housewife	1	5%
Multi-Occupation	1	5%
Unemployed	1	5%
Gender of the adolescent children		
Female	9	45%
Male	11	55%
Age of the adolescent children (years old)		
12	3	15%
13	6	30%
14	5	25%
15	6	30%

### Factors affecting adolescents’ physical activity during the COVID-19 pandemic

#### Self-determination and enjoyment to do physical activity

Overall, parents mentioned that their adolescent children did more physical activity before compared with during the pandemic. During pre-pandemic school days, children were doing physical activity in Physical Education lessons and many of them also did physical activity through participation in extracurricular sports activities at school or in sports clubs. A majority of mothers of boys reported that before the pandemic their sons were engaged in some forms of physical activity after school hours, such as playing football (soccer). Those activities changed significantly, especially during the beginning phase of the pandemic. Children spent most of the time at home due to social distancing and quarantining policy, and naturally became less active.

Self-determination seemed to be a key factor affecting adolescents’ physical activity during the pandemic. Adolescents who were more active before the pandemic were reported to maintain some form of physical activity during the pandemic, even without direct support from parents, thus showing forms of intrinsic motivation and self-determination [44]. An example of a more externally regulated form of self-determination is one overweight adolescent who was reported to do more physical activity during the pandemic as her awareness toward health increased and she had a goal to reduce weight. Another example is one male adolescent who used to be active in a sports club was also reported to maintain activity as he was determined to be an elite athlete for financial or economic reasons. This is also more externally regulated.

*“His dream is wanting to be a part of the national team”. “(He) asked how much salary of the national team?” (laughing) (I: laughing, yup). “It makes (him) so excited to volleyball, (he) loves it very much”* ***(P4, parents of 15 years old male children).***

Note: I = Interviewer; P = Participant

Enjoyment – a more intrinsic form of self-determination - also appeared to support adolescent’s self-determination in doing physical activity during the pandemic. Many parents reported that their adolescent child did many forms of sports as their hobby before the pandemic. Some months after the pandemic began, these adolescents were reported to continue doing their hobbies because of their enjoyment of the activities. Some adolescents were also reported to enjoy new physical activities during the pandemic, such as cycling with friends and family members. Some female adolescents were reported to enjoy participating in dance, such as following the popular K-Pop dance on YouTube.

*“Yes, (she watched) YouTube, and did dance for two (or) three songs (I: yup), dance like K-Pop is aerobic, right?”* ***(P1, parents of 13 years old female children).***

#### Support from others

Apart from self-determination and enjoyment, the way the adolescents did physical activity during the pandemic appeared to be influenced by support from others (i.e., family, friends, and teachers). During the beginning of the pandemic, many parents reported their adolescent children often do physical activity with other family members, such as cycling and morning walks, particularly during weekend days. The majority of parents also stated asking their children to help them with domestic-related physical activity, such as cleaning the house and gardening. Few parents facilitated their adolescent children to continue their training in sports clubs once they opened.

*“ … for example, her little sister when doing mm … dance then she was also following to dance in the living room”* ***(P13, parents of 14 years old female children)****.*

Moreover, having friends around the house supported adolescents to be active during the pandemic, especially for males. Many male adolescents were reported often going out for physical plays with their friends, such as playing soccer, because they felt more relaxed or not too afraid of the pandemic. Meanwhile, many mothers reported that their daughter felt reluctant to play outside of the house, even though they had friends around the house, as they were more afraid to be exposed to the COVID-19 virus. Adolescents also did a regular physical activity as a part of assignments from teachers for Physical Education lessons. While support from others seemed to facilitate adolescents to do more physical activity during the pandemic, the majority of mothers felt that their adolescents’ do more physical activity before compared with during the pandemic.

#### The availability of physical activity facilities and equipment

The majority of mothers reported that the proximity of physical activity facilities around the house, e.g., badminton field, free open space, affected their children’s involvement in physical activity. The options for physical activity remained limited as the majority of public sports facilities, such as swimming pools and sports fields, were closed. Few parents reported that having physical activity equipment at home, such as a treadmill and a stationary bicycle, made their children do more physical activity during the pandemic.

*“Yes, there is a badminton field near our house (I: Yup). Kids always played there in the afternoon. Sometimes he played there together (with his friends)”* ***(P8, parents of 12 years old male children)****.*

### Factors affecting adolescents’ sedentary behaviour during the COVID-19 pandemic

#### Educational demands during the pandemic

Before the pandemic, parents reported that children used screen-based devices for a quite limited time during school days. This was mainly because children spent most of the daytime at school, especially those participating in extracurricular activities. Most children did only engage in screen time in the late afternoon and/or evening for a limited time as they need to go to bed early to prepare for school the next morning.

However, parents reported a sharp increase in children’s screen time during the pandemic. Educational demands appeared to be one of the main factors driving this. All parents reported that doing online study at home makes their children access screen-based devices much more; their children were allocated many assignments and did study primarily by using screen-based devices.

***“****Screen use during the pandemic is for online study, she spent quite a lot of time in front of a screen because having a lot of assignments” “ … . in the beginning (of online study), in a day (she can do) 6 hours, only stopping for eating and praying”* ***(P10, parents of 14 years old female children).***

Interviews revealed that WhatsApp became the main platform for the school’s work. Children needed to access the school’s WhatsApp groups regularly to check updates, information, and assignments from their teachers. Children also used this platform to communicate or discuss assignments with their friends.

*“His Dad bought a smartphone for him because there are school’s WhatsApp groups that he used for...mm for (checking) school’s assignments. So, the assignments were sent to students through WhatsApp” (****P15, parents of 15 years old male children).***

Parents also reported that their children often accessed YouTube for educational purposes. Children look for educational information by watching videos on YouTube platforms or upload videos on YouTube as a part of the school’s assignment.

#### Psychological effects due to the impact of the pandemic

Children’s activities appeared to change drastically during the pandemic. The majority of parents indicated their children had some problems in arranging their activities. Having become accustomed to school routines, most children were reported to be confused about what to do at home. Because of requirements concerning social distancing and quarantining/isolating, children were encouraged to stay at home, especially in the early phase of the pandemic, which mostly closed their access to the outside world. Parents reported that this situation put some mental pressure on children and most of them then simply engaged for long periods of time in recreational screen use. This seemed to be to escape from the situation or to cope with boredom.

*“Doing nothing at home is impossible, right? (I: Yep) (she) then watched TV and used a smartphone to escape”* ***(P6, parents of 13 years old female children).***

*“Because of boredom (I: Yep) What else to do if not using a smartphone?” (laughing) (****P8, parents of 12 years old male children).***

Other psychological drawbacks that children felt during the pandemic seemed to be caused by educational demands, which resulted in some stress and emotional disturbance for adolescents. This also seemed to increase their need for recreational screen time. For recreational purposes, adolescents were using a smartphone much more than watching TV. Mothers mentioned that cartoon or animation programs had become their children’s favourites. Watching videos on the YouTube platform on a smartphone were also popular. This might be caused by various options that are offered and can be chosen based on the children’s interest and being accessible almost at any time. Many mothers of male adolescents mentioned that their son also used their smartphone heavily for playing games.

#### Devices and internet availability

The availability of the devices, such as a smartphone and television (TV), and internet access during the pandemic, influenced children’s screen time significantly. All parents reported that they facilitated their children’s learning during the pandemic by providing smartphones. Most parents reported having a TV at home and some provided a TV in the child’s bedroom. All parents also provided internet access; the majority of them facilitated internet access by buying internet quota from a mobile data provider; only some parents facilitated the use of Wi-Fi at home. Some parents reported that there is free Wi-Fi for the community near their house and that they sometimes permitted their children to go out to access this.

Although all parents expected that children used the device mainly for education, it cannot be denied that the majority of children also used the device a great deal for recreational and social purposes after or in between doing their school work. The availability of electronic media equipment, such as TV and smartphone, in the bedroom also facilitated children for more screen exposure.

*“At night (he) switched off (the TV) and sleep, but when I have slept the TV was on again” (laughing) (****P12, parents of 13 years old male children)****.*

#### Parental control

Mothers’ stories revealed that adolescent children were given more trust to manage their own time, thus they became more independent in doing so and choosing their screen-based activities. However, the majority of parents tried to apply some control of their adolescent children’s screen use by setting some rules, controlling facilities, and allocating non-screen activities.

Firstly, regarding rules, the majority of the parents tried to limit the duration of their children’s screen time, especially in the evening. Many mothers, who acted as the gatekeeper of children’s screen use in the family, reminded their children anytime they felt their child used screen-based devices for too long. No specific explanation about the duration of “too long” was evident as it appeared to be based mainly on parents’ feelings. However, many mothers admitted that there were times when their children were not willing to follow the rules and asked to use the devices for longer. Support from fathers to apply screen time rules seemed to facilitate a reduction in children’s screen time.

*“Mm … of course, there were barriers because sometimes depends on the mood. Kid’s mood if (she) wants to follow rules then (she) follow rules. If not in a good mood then, how to say it? Mm … kind of (pause) maybe now we call it to rebel, that happened, because the adolescent period is rebellious period, right?”* (***P10, parents of 14 years old female children).***

Concerning controlling facilities, many parents stated that they did not provide the use of Wi-Fi at home and provided a certain internet quota for a certain time to limit screen exposure in their children. These parents reported that these strategies were quite useful to make their children became more considerate in using their smartphone.

*“Yes, and we limit her in using it (internet quota) only for important matters. Because I checked the data usage. (Internet quota) should be used for certain days. If (it) often run out, it means not for that (education). So, they already knew and able to manage. “If I often run out internet quota then it means I did like this”, she already knew. I gave certain (internet) quota, like twenty thousand (Indonesian rupiah) for some days” (****P19, parents of 12 years old female children****).*

Lastly, regarding allocating non-screen activities, many parents reported that asking children to help with domestic tasks was quite useful to help their children be away from the smartphone, although temporarily. There was no clear difference in domestic tasks between female and male children. Many mothers stated that their male and/or female children helped them either to clean the house, wash clothes, dry clothes in the sun, iron, water plants, or helped them in the kitchen, such as cooking rice and boiling water.

Parents’ working location seemed to greatly influence their ability to control children’s screen time. Working outside the house during the day made many parents have less control over children’s activities and screen time. Parents who work outside the house admitted that they did not exactly know their children’s activities at home during the pandemic.

*“Kind of less attention (I: Yup) I work and go back late afternoon (I: Yup) Less control (toward him)” (****P11, parents of 14 years old male children****).*

#### Social facilitators

Family members and peers appeared to have a significant contribution to adolescents’ screen time during the pandemic. Many parents stated that their children often watch TV together with other family members, such as their siblings, during the day for their favourite programs. This was not the case before the pandemic as the adolescent children spent most of the time at school during the day.

Moreover, peers also influenced screen exposure in children. As children met their friends more rarely during the pandemic, it can be understood that they wanted to stay connected with their friends through social media platforms. WhatsApp and Instagram have become the most accessed social media by children. Overall, girls seemed to be more interested in using social media for chatting than boys.

*“So, she usually chats with her friends, like for preparation mm … what program they want to present? Like (pause) who will present for this program? (for) discussion, discussion forum” (****P10, parents of 14 years old female children)****.*

*“Yup, he is not keen on chatting with his friends, he rarely checked (WhatsApp)” (* ***P3, parents of 13 years old male children)*** *.*

Specific for the male children, having friends with the same interest in playing online games facilitate them to do more leisure screen time. Male children’s activities in playing online games seemed not only for leisure but also to facilitate social connection within their male friends.

*“There was influence from his friends. Everyone plays online games, they play (online games) together. His friends have Wi-Fi at their house, so they sometimes made an appointment, not gathering together in one place, but sometimes (they played together) at their own house, but already made an appointment” (****P20, parents of 15 years old male children).***

## Discussion

This study aimed to investigate mothers’ perceptions of underlying factors affecting changes in physical activity and sedentary behaviour in adolescents during the COVID-19 pandemic. We identified three themes regarding factors associated with changes in physical activity during the COVID-19 pandemic. Firstly, this study found that self-determination and enjoyment seemed to be one of the main factors affecting physical activity positively in adolescents during the pandemic. Self-determination to do physical activity may be influenced by the need to grow [45], such as wanting to be an elite athlete, or because of having intrinsic motivation [45], such as doing physical activity for enjoyment. Enjoyment appeared to play an important role in supporting self-determination. Being highly self-determined to do physical activity may be simply started from the enjoyment that someone felt when engaged in physical activity. Previous studies have found that enjoyment is a significant factor for maintaining participation in physical activity [46, 47], although conceptually, the construct of enjoyment is sometimes complex (see [48]).

However, few adolescents clearly showed that their self-determination to do physical activity was also influenced by incentives concerning what they want to achieve. This is consistent with Bandura’s social cognitive theory which posits that behaviour is determined by expectancies and incentives, such as obtaining an ‘ideal’ body shape and gaining economic benefits [49]. Interventions for increasing adolescent physical activity may need to consider how to develop participant’s self-determination by ensuring that the program offers enjoyment and emphasizes the incentives that participants can get, such as good health status and satisfying physical appearance.

Secondly, in line with previous studies [50–53], we revealed that support from family, friends, and teachers seemed to affect adolescents’ physical activity during the pandemic. A recent longitudinal study also found that parental support was positively correlated with MVPA in youth [54]. As Granich et al. [50] pointed out, role modelling and reinforcement from parents have become important factors for influencing children’s behaviour. Parents’ support for adolescents to join sports clubs, such as providing transportation and sports equipment, has facilitated adolescents to be physically active. Meanwhile, siblings facilitated adolescent’s physical activity by increasing opportunities for adolescents to engage with different types of physical activity, such as free play [50].

Peers have also been found to be important facilitators for adolescents’ physical activity during the pandemic. For example, adolescents tended to be more involved in greater intensity of physical activity when getting together with their peers [47, 52, 55]. Friends provided support by co-participation, role-modelling, giving verbal encouragement, and increasing enjoyment in doing physical activity [46, 47]. Moreover, teachers provided supports on physical activity through Physical Education, both before and during the pandemic. Taken together, physical activity interventions in adolescents should consider involving family, friends, and teachers in implementing the programs.

Lastly, regarding the availability of physical activity facilities and equipment, this study revealed that these factors supported adolescents in doing more physical activity during the pandemic. Aligned with this finding, a previous systematic review found that there was an association between physical activity behaviour and the presence of physical activity equipment at home [56]. Increasing the availability and accessibility of physical activity facilities in open spaces close to community settlements may trigger adolescents to do more physical activity and reduce their overall sedentary behaviour.

We generated five themes concerning sedentary behaviour. Firstly, this study found that demands for online-based learning during the COVID-19 pandemic have caused a significant increase in adolescents’ screen time. The reasons children used more screen-based devices for online study is obviously associated with the creation of much greater demands for this type of delivery of teaching and learning during the COVID-19 pandemic. Moreover, it may be partly explained by using the concept of extrinsic motivation, such as when adolescents completed assignments to avoid punishment or low marks [57]. Furthermore, it may be caused by the need to fulfill basic psychological needs, especially competence [57]. As one of the primary psychosocial tasks that adolescents must achieve is ‘to measure up’ – that is to develop competence and find ways to achieve the goal [58]- it has become the nature of adolescents that they want to show their capability, skills, and competence, such as by performing well in finishing school assignments. These necessitate studying through the use of internet-connected devices.

While adolescents spent much greater screen time for educational purposes during the pandemic, this might not necessarily cause the adverse effects as if the screen time were used for recreational purposes. Sanders et al. [59] found that types of screen use moderated outcomes of screen use. For example, educational screen time had a positive correlation with educational outcomes and higher persistence, with no negative consequences for health outcomes. Nevertheless, consistent with international guidelines, it is suggested that adolescents break up prolonged sitting when studying to avoid any health risks that may arise from static postures and lack of movement.

Secondly, this study found that adolescents did experience some negative psychological outcomes, such as stress and boredom, during the pandemic. These seemed to facilitate them consuming more recreational screen time. This is consistent with previous studies that found that the COVID-19 pandemic has created mental health problems in adolescents, including high anxiety rates, concentration difficulties, depression, and reduced happiness [60–62]. Stierlin et al. [63] found a similar result that youth with more mental health problems tended to have greater screen time.

The root cause of psychological problems that were experienced by adolescents during the pandemic may come from sleep deprivation [64]. Suggestions to stay indoors during the pandemic may have caused a reduced exposure to sunlight, which is essential in maintaining a regular sleep routine [23]. Moreover, the excessive use of electronic media, especially for social media or internet use and playing online games, had been found to have an association with shorter sleep duration and decreased sleep quality [65, 66], especially close to bedtime. Screen use that involves active engagement before bedtime may disturb the ability to sleep as that activity may cause physiological and psychological arousal [65].

In addition, physical distancing and quarantining measures that were implemented during the pandemic have caused a significant reduction in physical activity and a clear increase in electronic media use [23, 61, 62, 67]. Participation in the physical activity itself is important for maintaining good mental health [68, 69]. Meanwhile, excessive recreational screen time has been associated with poor psychological well-being [70–72].

However, not all recreational screen time causes negative effects on young people. When done appropriately and moderately, it may bring positive effects as it can boost mood and provide enjoyable recreation. This is consistent with previous studies that recreational screen time may result in both positive and negative effects, depending on how children use it [73]. Screen use might provide social benefits by enabling communication within friends, provide emotional satisfaction and relaxation, such as from watching humorous content, but may also be detrimental to emotional health, such as causing depression from experiencing cyberbullying [73]. Given the ubiquitous nature of contemporary screen use, it is important to recognise the positive as well negative uses. Strategies to reduce recreational screen time, therefore, should also include education on healthier recreational screen time to minimize adolescents from getting negative effects from screen-related activities. This approach can be incorporated into the most popular programs and platforms for adolescents, such as cartoon and YouTube, and should improve awareness of the psychological, behavioural, and physiological effects of excessive screen time [50] as well as the benefits of physical activity.

Thirdly, consistent with previous studies [50, 56, 74, 75], we identified that the availability of screen-based devices and internet access has influenced adolescents’ screen time significantly. Having internet access always available on a smartphone, either from a mobile data provider or through Wi-Fi, appeared to increase adolescents’ opportunity to surf the internet anytime and anywhere. A recent study by Thomas et al. [73] showed a similar finding that internet access availability facilitated adolescents to spend more time on screens.

Previous experimental studies found that limiting accessibility toward screens has successfully reduced screen exposure in children [56, 75]. Thus, future intervention studies aiming to reduce adolescents’ screen time may consider limiting accessibility toward electronic media equipment at home, such as limiting the permitted duration at certain times of the day [76] and allocating specific rooms to use the devices.

Furthermore, our study revealed that parental control is likely to influence the length of adolescents’ screen time. A previous study found similar results whereby rules and restrictions for using electronic media moderated the duration of children accessing screen media [50]. Our study also found that mothers tend to be the dominant gatekeepers of adolescents’ screen use. Therefore, it may be important to involve mothers, as well as other family members, in interventions to modify adolescents’ screen time. Promotion of current guidelines on sedentary behaviour in adolescents, such as the 2020 WHO guidelines on sedentary behaviour [77], is also important to increase parents’ awareness on how to guide screen use for their children. Other guidance also exists [78]. Nevertheless, the process of modifying behaviours in adolescents should also consider other internal factors within adolescents that motivate them to accept and change their behaviour [50], such as self-efficacy. Involving adolescents in such decisions is important.

Lastly, aligned with a previous study [50], the current study suggests that family members and peers are found to be social facilitators that affect screen use in adolescents. Peers appeared to influence adolescents’ screen time through social media and online games. Adolescents seemed to try to stay connected with their friends through these platforms and to fulfill one of their basic psychological needs, that of social relatedness [57], and the psychosocial task to fit in – “to find comfortable affiliations and gain acceptance from peers” [58].

WhatsApp and Instagram have become popular social media platforms accessed by adolescents. Therefore, future interventions to promote physical activity and to reduce excessive sedentary behaviour may need to consider using these applications and other recent popular applications among adolescents in implementing their programs. While it may seem ironic to use screen-based platforms to change screen use, it may be a suitable and practical strategy if used properly (see [76]).

Regarding adolescents’ active involvement in playing online games, it may be important to make recommendations on the types of games that may facilitate physical activity, psychological health, social interaction, and collaboration with others [79]. However, it is important to bear in mind that excessive gaming may cause detrimental effects for adolescents, including excessive sedentary behaviour, sleep deprivation, physical aggression, depression, and academic problems [80]. Therefore, providing education on appropriate gaming for adolescents seems to be important.

### Strengths and limitations

A strength of this study is that it adds evidence to the limited qualitative literature examining factors likely affecting changes in adolescents’ physical activity and sedentary behaviour during the COVID-19 pandemic, especially in LMICs. The heterogeneous nature of the participants’ demographic background has enabled the researchers to gather a range of stories, opinions, and experiences which add to the richness of the data. Moreover, the semi-structured interview format allowed the interviewer to probe for more information and gather deeper insights on the interview topics. Having personal connections with some participants and sharing similar cultural backgrounds with all participants also have helped the interviewer in building rapport during interviews, which may help in facilitating the participants to share their true stories.

Limitations of this study include the sample from one region of Indonesia, therefore findings may not be true for other regions in Indonesia and other countries. Nevertheless, it is consistent with the objective of the qualitative study that has no aim to generalise its findings to other populations. Moreover, it is undeniable that some participants may feel some hesitation in telling all stories due to social desirability bias. Participants may also have limitations in providing immediate responses to some questions and forget some details of their stories and views.

## Conclusions

This study provides new insights on possible underlying factors affecting changes in Indonesian adolescents’ physical activity and sedentary behaviour during the COVID-19 pandemic. Overall, adolescents appeared to become less active and more reliant on screen-based devices, either for educational or recreational purposes. WhatsApp, Instagram, YouTube, and cartoons were the most popular social media and programs. Future studies and policymakers should account for these findings when considering interventions and policies.

Future studies aiming at increasing physical activity and reducing sedentary behaviour in adolescents should consider increasing parents’ and adolescents’ awareness of current activity guidelines, the risks of excessive sedentary behaviour, and providing education on healthier recreational screen time. Involving parents, peers, and teachers, as well as the adolescents themselves, in intervention programs may provide substantial benefits. Moreover, physical activity intervention programs for adolescents should consist of activities that are enjoyable and satisfying, with an increase in the number and range of physical activity facilities and equipment accessible near to community settlements being important.

## Supplementary Information

**Additional file 1: Table S1.** Standards for Reporting Qualitative Research Checklist.

## Data Availability

The data (anonymized/deidentified) that support the findings of this study are available from the corresponding authors on reasonable request.

## Abbreviations

WHO: World Health Organization
MVPA: Moderate-to-vigorous physical activity
COVID-19: Severe acute respiratory syndrome coronavirus 2 (SARS-CoV-2)
LMICs: Low-to-middle income countries
SRQR: Standards for Reporting Qualitative Research
TV: Television
I: Interviewer
P: Participant
