# Supplementary material for: Adolescents’ physical activity and sedentary behaviour in Indonesia during the COVID-19 pandemic: a qualitative study of mothers’ perspectives
Source: BMC Public Health. 2021 Oct 15;21:1864. doi: 10.1186/s12889-021-11931-1 (PMC8519321; doi:10.1186/s12889-021-11931-1)
Supplement: Supplementary file 1 — Additional file 1: Table S1. Standards for Reporting Qualitative Research Checklist. [file 12889_2021_11931_MOESM1_ESM.docx]

Table S1. Standards for Reporting Qualitative Research (SRQR) Checklist

| **No** | **Topic** | **Line(s)** |
| --- | --- | --- |
| S1 | Title | 1 – 2 |
| S2 | Abstract | 12 – 33 |
| S3 | Problem formulation | 70 – 91 |
| S4 | Purpose or research question | 92 – 100 |
| S5 | Qualitative approach and research paradigm | 104 – 114 |
| S6 | Researcher characteristics and reflexivity | 188 – 207 |
| S7 | Context | 119 – 121 |
| S8 | Sampling strategy | 116 – 138 |
| S9 | Ethical issues pertaining to human subjects | 634 – 637 |
| S10 | Data collection methods | 140 – 159 |
| S11 | Data collection instruments and technologies | 149 – 150 |
| S12 | Units of study | 226 |
| S13 | Data processing | 162 – 163 |
| S14 | Data analysis | 164 – 186 |
| S15 | Techniques to enhance trustworthiness | 168 – 171, 194 – 207 |
| S16 S17 | Synthesis and interpretation  Links to empirical data | 209 – 454 |
| S18 | Integration with prior work, implications,  transferability, and contribution(s) to the field | 456 – 591 |
| S19 | Limitations | 603 – 608 |
| S20 | Conflicts of interest | 646 – 647 |
| S21 | Funding | 649 – 650 |
